# An application-specific image processing array based on WSe_2_ transistors with electrically switchable logic functions

**DOI:** 10.1038/s41467-021-27644-3

**Published:** 2022-01-10

**Authors:** Senfeng Zeng, Chunsen Liu, Xiaohe Huang, Zhaowu Tang, Liwei Liu, Peng Zhou

**Affiliations:** 1 State Key Laboratory of ASIC and System, School of Microelectronics, Fudan University, 200433 Shanghai, China; 2 Frontier Institute of Chip and System&Qizhi Institute, Fudan University, 200433 Shanghai, China; 3 Shanghai Frontier Base of Intelligent Optoelectronics and Perception, Institute of Optoelectronics, Fudan University, 200433 Shanghai, China

**Keywords:** Electronic devices, Applied physics

## Abstract

With the rapid development of artificial intelligence, parallel image processing is becoming an increasingly important ability of computing hardware. To meet the requirements of various image processing tasks, the basic pixel processing unit contains multiple functional logic gates and a multiplexer, which leads to notable circuit redundancy. The pixel processing unit retains a large optimizing space to solve the area redundancy issues in parallel computing. Here, we demonstrate a pixel processing unit based on a single WSe_2_ transistor that has multiple logic functions (*AND* and *XNOR*) that are electrically switchable. We further integrate these pixel processing units into a low transistor-consumption image processing array, where both image intersection and image comparison tasks can be performed. Owing to the same image processing power, the consumption of transistors in our image processing unit is less than 16% of traditional circuits.

## Abstract (web summary)

Reducing circuit redundancy represents a priority for the scalability of parallel computing hardware. Here, the authors report the realization of pixel processing units consisting of single 2D WSe_2_ transistors implementing electrically-switchable logic functions. This strategy enables the fabrication of an image processing array with ~16% transistor consumption compared to traditional circuits.

## Introduction

As artificial intelligence technology evolves, the amount of data that computers need to process has greatly increased, especially image and video data. To deal efficiently with increasingly massive data, adopting parallel computing hardware is the market trend. Parallel computing has been considered “the high end of computing” and is more suitable for hardware acceleration and image processing applications^1,2^. In commercial neural network hardware acceleration applications, the widely used NVIDIA graphics processing unit^3^ and Google Tensor processing unit^4^ are all based on parallel computing architecture. For image data, multiple pixels are parallelly processed for efficiency. According to different computing tasks, different function modules need to be involved in each unit; for example, the logic *AND* function is applied to find the intersection of images, while the logic *XNOR* function is applied to compare the similarity of images. This requires each pixel processing unit to contain multiple functional modules on the hardware level, which are switchable according to task requirements. The existing technical path has a very high area redundancy because each functional module of the pixel processing unit is physically implemented by different circuits and the function selection depends on additional control circuits (such as multiplexer). The highly parallel computing process further increases the circuit redundancy because the complex pixel processing units need to be repeated in an array.

Unlike bulk materials, two-dimensional (2D) materials have atomic-level thickness and abundant electronic characteristics, which has potential in the design of emerging electronic devices^5–7^. Many studies have been performed to build logic circuits using 2D materials, such as n-type metal-oxide-semiconductor (NMOS) logic circuits^8–11^ and various reconfigurable logic gates schemes^12–18^. These works have remarkable progress in functional integration by utilising the characteristics of 2D materials. To promote 2D material devices to practical parallel computing applications, there are still important challenges to determine, such as electrical switchable logic functions and compact device structures. Until now, a compact transistor structure that has multiple electrical switchable logic functions has been missing. A compact structure that only has the requisite terminals for power supply and input/output can guarantee the area efficiency of circuits. Multiple electrical switchable logic functions can satisfy different task processing demands. Such a device has the potential to simulate the functions of a single-pixel computing unit to handle different graphics tasks.

In this work, we experimentally demonstrate an image processing array by 2D material WSe_2_. Through drain voltage regulation on the carrier injection barrier, the logic function is switchable between *AND* and *XNOR* in a single transistor without additional terminal or multiplexer circuits, which means that the single device is qualified to the pixel processing unit. This single transistor pixel processing unit greatly decreases the consumption of transistors in logic circuits (1 transistor implements logic *AND* and *XNOR*) compared to the NMOS logic family (2 transistors for logic *AND*, 8 transistors for logic *XNOR*, and additional multiplexer circuits). Assembling these WSe_2_ pixel processing units into an array, the image processing array can handle different graphic processing tasks, such as finding the intersection or similarity of images. At the same processing power, the transistor consumption of our image processing array is <16% of the traditional scheme, which has the potential to remove the circuit redundancy issue in parallel computing.

## Results

### Low transistor consumption image processing array

For image processing, each pixel data point is processed by a pixel processing unit. Physically using different circuits to implement various logic gates and adding additional multiplexers to switch functions will increase the redundancy of the circuit. According to the two-surface-channel (TSC) working mechanism^12^, logic computing can be performed in a single double-gated transistor. Utilising the voltage-modulated barrier effect, we have successfully implemented a pixel processing unit by only a single TSC WSe_2_ transistor.

**Fig. 1:**
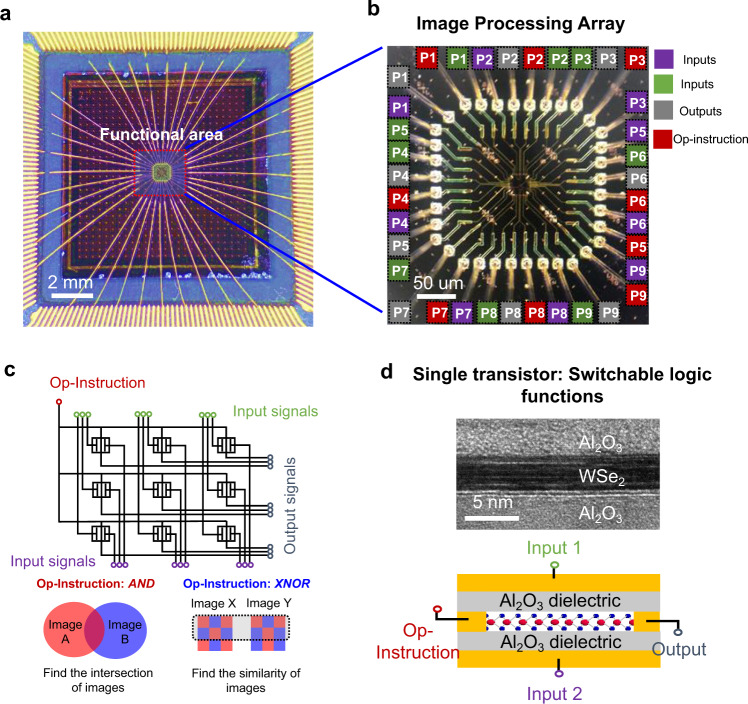
Image processing array with switchable functions. **a** Macroscopic image of the bonded device on the carrier, which consists of 3 × 3 pixels, scale bar: 2 mm. **b** The optical image of the TSC image processing pixel array, scale bar: 50 μm. We use P1-P9 to mark the ports of each pixel unit. The input 1, input 2, output and Op-instruction ports are coloured by purple, green, grey and red respectively. **c** Schematic circuit diagram of the pixel processing array. With different Op-Instruction inputs, image intersection and comparing functions are implemented. **d** Top part: The cross-sectional high-resolution TEM image. The scale bar is 5 nm. Bottom part: Schematic diagram of the single-pixel processing unit. The drain and source of the device serve as the OP-Instruction and output ports, the top gate and bottom gate serve as input 1 and input 2. With the Op-instruction signal input, a single transistor can perform switchable logic functions.

Figure 1a is the packaged image processing array based on the TSC WSe_2_ transistors, and Fig. 1b is the functional area where the terminals are marked in different colours according to their electrical connections (green is input1, purple is input2, red is Op-Instruction and grey is output). The array consists of 3 × 3 TSC WSe_2_ transistors, and all the devices are encapsulated with an Al_2_O_3_ dielectric. The detailed fabrication process and energy dispersive spectroscopy (EDS) analysis are provided in Supplementary Notes 1 and 2. In addition, we demonstrated the scalability of the process in Supplementary Note 3 by using thin films of a large-area CVD film. The devices have an adjustable switching ratio to adapt to different operating conditions (Supplementary Note 4). Figure 1c shows the circuit layout and its image processing abilities. The cross-sectional transmission electron microscopy (TEM) image and the schematic device structure of one of the pixel computing units are shown in Fig. 1d. The top and bottom gates function as inputs, the source terminal is chosen as the output (current signal) and the drain serves as the Op-Instruction (voltage signal). It is worth noting that there is no additional terminal induced to regulate the logic function of the device; therefore, the area efficiency has not been sacrificed for multiple logic functions.

### Mechanism of electrically switchable logic functions

Different from unipolar n-type semiconductor MoS_2_ with sulfur vacancies and strong Fermi level pinning near the conduction band^19^, WSe_2_, as an ambipolar semiconductor, has been demonstrated to effectively shift the Fermi level between the valence band and conduction band under the application of an external field^20^. This makes it possible for both holes and electrons to act as carriers in the WSe_2_ channel when a different voltage is applied. Therefore, WSe_2_ was selected as the channel material because the WSe_2_ transistor should have the potential to show various logic functions.

**Fig. 2:**
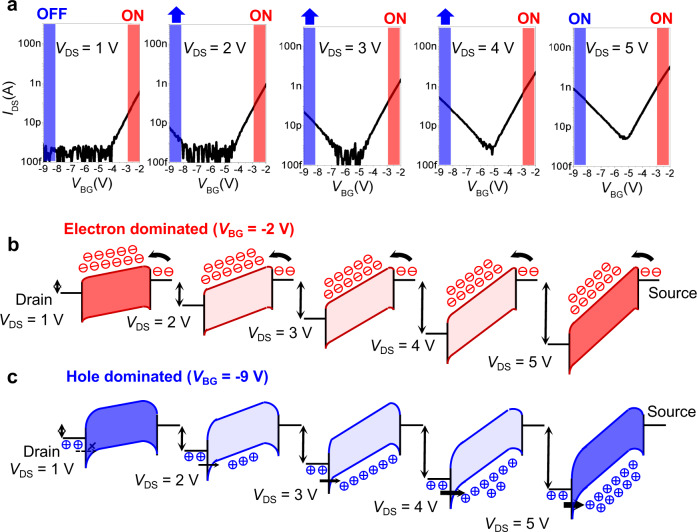
The mechanism of switchable logic functions by voltage control. **a** The transfer characteristic curves of *I*_DS_-*V*_BG_ at different *V*_DS_ from 1 V to 5 V. The blue shaded area represents the hole conduction dominated region (*V*_BG_ = −9V), the red shaded area represents the electron conduction dominated region (*V*_BG_ = −2V) (The *V*_S_ is fixed to zero voltage, adjust *V*_DS_ only by changing *V*_D_). **b** The band diagram of the electron-dominated region under different *V*_DS_ (The height of the black arrow represents *V*_DS_). The electrons are injected into the channel across the barrier between the source and the channel. *V*_DS_ has little effect on the conduction current. **c** The band diagram of the hole-dominated region under different *V*_DS_. The holes tunnel through the barrier between the drain and the channel and inject into the channel. As the *V*_DS_ increases from 1 V to 5 V, the tunnelling efficiency increases, increasing the hole current.

First, we studied the modulation effect of the drain-source voltage (*V*_DS_) on the polarity of the TSC WSe_2_ transistor. As Fig. 2a shows, the characteristic drain-source current (*I*_DS_)-bottom gate voltage (*V*_BG_) transfer curves are used to calibrate the basic performance of the device (the TG is no input). At different *V*_DS_ values, the majority carrier in the channel materials significantly changes from only electrons to both electrons and holes. To analyse the mechanism, the band structures of the device in the electron-dominated region (*V*_BG_ = −2 V) and the hole-dominated region (*V*_BG_ = −9 V) at different *V*_DS_ values were plotted. For identification, we colour-coded the carrier types, and the blue and red regions are hole- and electron-dominated carriers, respectively.

Figure 2b shows the band diagram development of the electron-dominated region with increasing *V*_DS_. Because the Al_2_O_3_ deposition process has an n-doping effect on the WSe_2_ channel, the device exhibits the n-type at a low drain voltage. As *V*_DS_ increases, the electron current intensity does not show a noteworthy change. From the corresponding band diagram, we can see the electrons injected into the channel from the source electrode. The electron injection barrier (source/channel interface) is regulated by the gate voltage, and the drain voltage has little impact on this barrier because the source is grounded. Therefore, the electron-dominated region is not greatly affected by *V*_DS_. Figure 2c shows the band diagram development of the hole-dominated region with increasing *V*_DS_. In this case, the hole current intensity changes as *V*_DS_ increases. Different from the electron-dominated region, the hole current intensity is determined by the barrier at the drain/channel interface and regulated by both gate voltage and drain voltage. A large *V*_DS_ will thin the triangle hole barrier and distinctly increase the injection number of holes from the drain electrode into the channel.

**Fig. 3:**
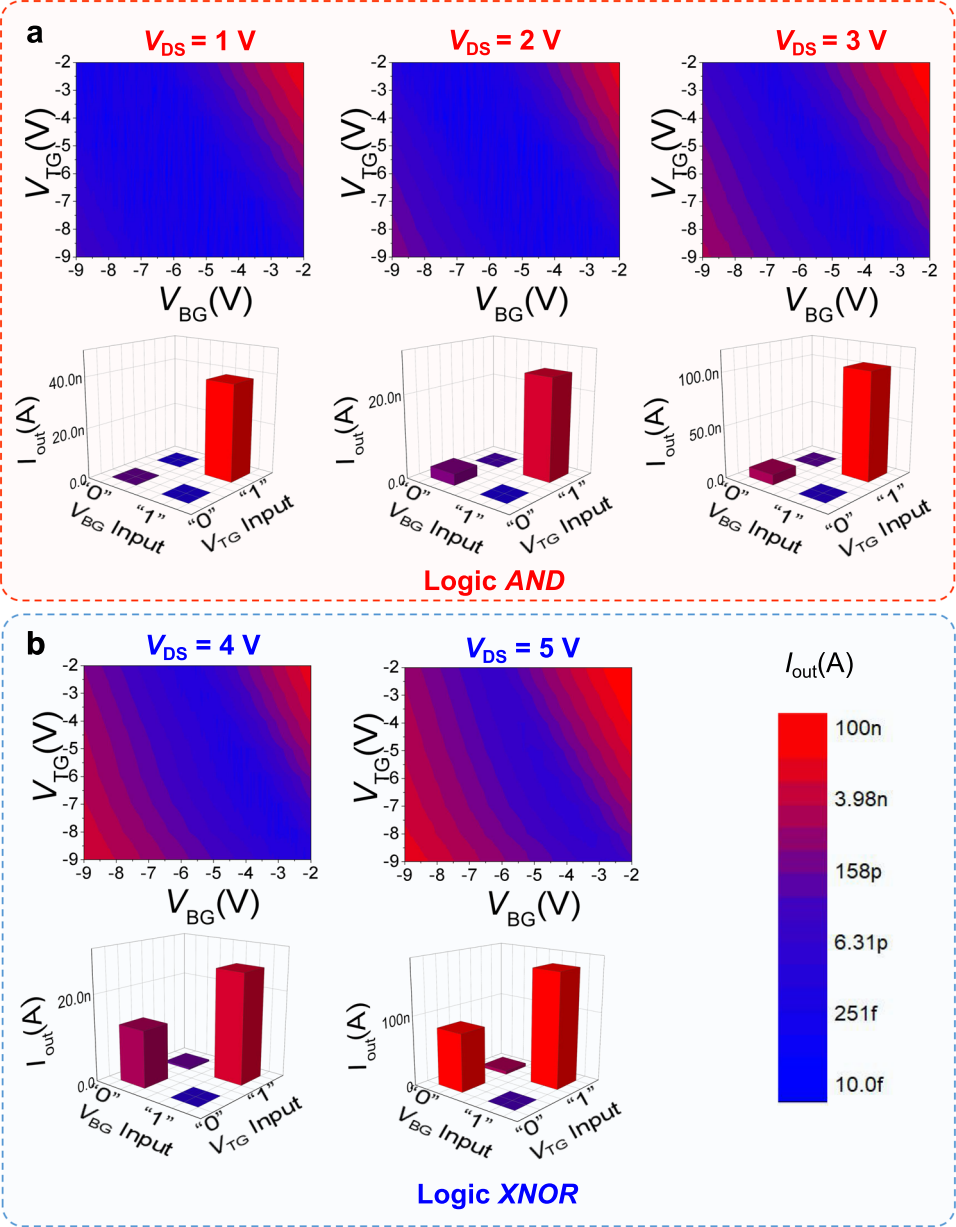
The logic computing behaviours of single processing pixel. Drain current *I*_out_ is mapped as a function of *V*_TG_ and *V*_BG_ under different Op-instruction of *V*_DS_ from 1 V to 5 V. Taking −2 V and −9 V as input “1” and “0” of *V*_TG_ and *V*_BG_, the logic behaviours are shown by the current bar of *I*_out_. At low operating voltages (1 V) of Op-instruction, a single processing pixel realises *AND* logic, while at high operating voltages (5 V), *XNOR* logic is implemented. **a** The output current and logic behaviours under *V*_DS_ from 1 V to 3 V, devices show logic *AND*. **b** The output current and logic behaviours under *V*_DS_ from 4 V to 5 V, devices show logic *XNOR*.

We have already demonstrated that the hole current of the WSe_2_ channel (encapsulated with Al_2_O_3_) could be modulated by the drain voltage. This useful feature could be utilised in the TSC transistor to realise electrically switchable logic functions without additional control terminals. In Fig. 3, we carried out voltage scanning from −9 V to −2 V for both the top gate and bottom gate to obtain the output current map. The horizontal and vertical axes represent *V*_BG_ and *V*_TG_, respectively (the data came from the first-pixel processing unit in the array). The test data of the remaining units are provided in Supplementary Note 6. According to the principle of the TSC transistor, the output current is jointly controlled by two gates. When the two gate input voltages are “0”(−9 V) and “1”(−2 V) respectively, the TG and BG compete with each other for the control of the channel potential and conduction. The “competition” leads to an intermediate channel potential that makes the Schottky barrier high for both electron & hole, and the channel is turned off. When the two gate input voltages are both “0” or “1”, the conductivity of the channel is controlled by *V*_DS_. When *V*_DS_ is increased from 1 V to 3 V, the left-bottom corner (hole-dominated region) representing the output current is much less than the right-top corner (electron-dominated region) that represents the output current, and the corresponding logic function of the TSC WSe_2_ transistor is the *AND* gate (Fig. 3a). When *V*_DS_ is above 3 V, the hole current is nearly the same as the electron current, and the corresponding logic function of the device is the *XNOR* gate (Fig. 3b).

Taking the *V*_DS_ as Op-Instruction and *I*_DS_ as the output current signal, the transistor logic function can be switched by *V*_DS_ without additional control terminals or multiplexer circuits. We can see that as the Op-Instruction signal changes from 1 V to 5 V, the basic functions of this single-pixel computing unit are switched from *AND* to *XNOR*.

### Analysis of transistor consumption

To visualise the consumption of transistors in various logic circuits, the normalised transistor consumption is used to measure the number of transistors required in the different logic gates in terms of the NMOS-based logic circuits. According to the output signal type, the logic gate can be divided into *V* (voltage input)–*I* (current output) logic and *V* (voltage input)–*V* (voltage output) logic. Adding a depletion-load transistor, V-I logic can always be transformed into *V*–*V* logic; therefore, transistor consumption computing is based on *V*–*V* logic.

As the baseline, the transistor number of NMOS logic is 9 (*XOR*), 3 (*NAND*) and 3 (*NOR*). The transistor consumption calculation method is given by

1 $${{{{{\mathrm{Transistor}}}}}}\,{{{{{\mathrm{consumption}}}}}}=\frac{{T}_{XOR}+{T}_{NAND}+{T}_{NOR} ({{{{{\mathrm{based}}}}}}\,{{{{{\mathrm{on}}}}}}\,{{{{{\mathrm{emerging}}}}}}\,{{{{{\mathrm{logic}}}}}})} {{T}_{{XOR}}+{T}_{{NAND}}+{T}_{{NOR}}({{{{{{\mathrm{based}}}}}}}\,{{{{{{\mathrm{on}}}}}}}\,{{{{{{\mathrm{NMOS}}}}}}}\,{{{{{{\mathrm{logic}}}}}}})}\times {{{{{\mathrm{PG}}}}}}$$

where *T*_*XOR*_, *T*_*NAND*_, and *T*_*NOR*_ indicate the number of transistors required to implement logic *XOR*, *NAND*, and *NOR*, respectively, and PG represents the number of planar gates in a single transistor.

Table 1 is the transistor consumption summary of various logic transistor technologies. In Supplementary Note 5, we analysed the circuit structure of each work and the calculation method of transistor consumption in detail. To solve the circuit redundancy issue in parallel computing, multiple logic functions should be implemented by as few transistors as possible. Notably, the addition of more multiplexer or control gate terminals to switch logic functions will also induce extra circuit redundancy. As Table 1 shows, a single compact TSC WSe_2_ transistor can implement a pixel processing unit, and the transistor consumption is <16% of the NMOS logic baseline.

**Table 1:** The transistor consumption summary of various logic transistor technologies.

Reference	Planar gate number	Transistor Number (V-V Logic)					Control signal	Computing method of transistor consumption	Transistor consumption
		*XOR*		*NAND*		*NOR*			
*The NMOS-based logic gate*									
Standard Si NMOS	1	9	3		3		None	$\frac{ \left(9\right.({XOR})+3({NAND})+3({NOR})} { \left(9\right.({XOR})+3({NAND})+3({NOR})}\times 1$	1
MoS_2_ NMOS [8]	1	9	3		3			$\frac{\left(9\right.({XOR})+3({NAND})+3({NOR})}{\left(9\right.({XOR})+3({NAND})+3({NOR})}\times 1$	1
MoS_2_ NMOS [9]	1	9	3		3			$\frac{\left(3\right.({NAND})+3({NOR})}{\left(3\right.({NAND})+3({NOR})}\times 1$	1
MoS_2_ NMOS [10]	1	9	3		3			$\frac{\left(9\right.({XOR})+3({NAND})+3({NOR})}{\left(9\right.({XOR})+3({NAND})+3({NOR})}\times 1$	1
Carbon nanotube NMOS [11]	1	9	3		3			$\frac{\left(3\right.({NAND})+3({NOR})}{\left(3\right.({NAND})+3({NOR})}\times 1$	1
*Functions tunable logic gate*									
BP transistor regulated by polar gate [13]	2	5	4 (*NAND* & *NOR*)				Voltage	$\frac{ \left(5\right.({XOR})+4({NAND}\,\& \,{NOR})}{\left(9\right.({XOR})+3({NAND})+3({NOR})}{{\times }}2$	1.2
WSe_2_ transistor regulated by polar gate [14]	2	5	4 (*NAND* & *NOR*)				Voltage	$\frac{\left(5\right.({XOR})+4({NAND} \&\,{NOR})}{\left(9\right.({XOR})+3({NAND})+3({NOR})}{{\times }}2$	1.2
MoS_2_ transistor regulated by trapped charges [15]	1	9 (*XOR* & *NAND*)			/		Voltage	$\frac{9({XOR}\,{{\& }}\,{NAND})}{\left(9\right.({XOR})+3({NAND})}\times 1$	0.75
WSe_2_ transistor regulated by polar gate [18]	2	/	2 (*NAND* & *NOR*)				Voltage	$\frac{\left(2\right.({NAND}\,\& \,{NOR})}{\left(3\right.({NAND})+3({NOR})}\times 2$	0.66
Transistor with channel materials (MoS_2_, WSe_2_, BP) [17]	1	2	2		2		/	$\frac{\left(2\right.({XOR})+2({NAND})+2({NOR})}{\left(9\right.({XOR})+3({NAND})+3({NOR})}\times 1$	0.4
MoS_2_ transistor regulated by light [12]	1	/	2 (*NAND* & *NOR*)				Light	$\frac{\left(2\right.({NAND}\,\& \,{NOR})}{\left(3\right.({NAND})+3({NOR})}\times 1$	0.33
**This work**	1	2 (*XOR* & *NAND*)			/		Voltage	$\frac{\left(2\right.({XOR}\,\& \,{NAND})}{\left(9\right.({XOR})+3({NAND})}\times 1$	0.16

Planar gate number: The number of planar gates in a single transistor.

Transistor Number(V-V Logic): The number of transistors required to implement the different logic function (in voltage-input-voltage output logic).

Control signal: The signal used to switch logical functions.

Computing method of transistor consumption: The formula for calculating transistor consumption.

Transistor consumption: Ratio of the number of transistors consumed to conventional logic circuits.

In different work, we enumerate the number of transistors required to realise *XOR*, *NAND* and *NOR* logic functions under different technology paths, and calculate their transistor consumption to realise logic functions. The detailed analysis and computing method of transistor consumption are discussed in Supplementary Note 5.

### Demonstration of image processing tasks

Based on this TSC WSe_2_ transistor, we fabricated a 3 × 3 image processing array. We demonstrate two different kinds of image processing tasks (finding the intersection or similarity of two images) in the same low transistor consumption hardware. The input images are simplified to a binary pixel, input signals “0” (−9 V) and “1” (−2 V) are used to represent the pixels of low grey level and high grey level and the data of two images are input from the top gate and bottom gate, respectively. The drain voltage is used as the Op-Instruction, and the source current is selected as the output signal. When the Op-Instruction imposes *AND* instruction (1 V), the array realises the function of image intersection. When the *XNOR* instruction (5 V) is imposed, the array can compare the similarity of the two images. Next, we will explain the specific implementation method.

**Fig. 4:**
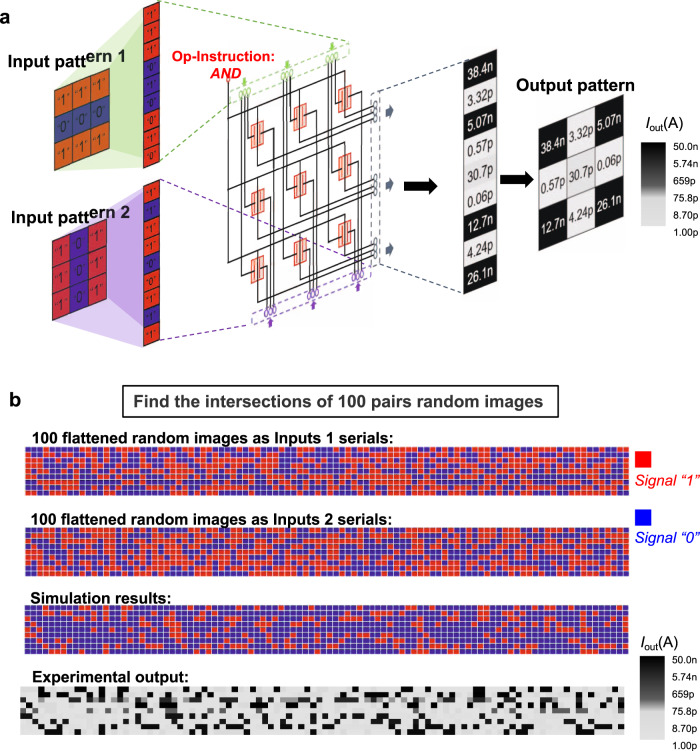
Demonstration of the images intersection function based on logic *AND*. **a** Diagram of the working process of images intersection function. Two 3 × 3-pixel patterns are input in parallel from each of the two input ports of the pixel process units. The current of each pixel serves as the output signal. **b** A total of 100 pairs of 3 × 3-pixel graphs are randomly generated, the signal “1” and “0” refer to −2 V and −9 V respectively. All the random patterns are expanded in the manner shown in **a**, the 3 × 3-pixel patterns are represented in the way of 9 × 1, and 100 groups of graphs are combined. Then the flattened input patterns serials are shown as the first and second rows. The third row shows the truth table of the flattened computing results. The red blocks and blue blocks represent the output “1” and “0” (computing results of logic *AND*). The fourth row is the actual output current value which is consistent with the truth table.

Figure 4 demonstrates how to find the intersection of two images in our array. As shown in Fig. 4a, a 3 × 3 pixel graph can be encoded as a 9*1 binary array, as we defined earlier. Two images are inputted to the top gate and bottom gate of each pixel computing unit. All the drain electrodes of each pixel computing unit are set to 1 V (Op-Instruction: *AND*), and the sampling currents are used as the output signals. Once the input and Op-Instruction have been given, the computing results will synchronise the output. Through the colour bar, we can obtain the intersection parts of the two input graphs. In addition, we randomly generated 100 groups of images (Row 1 and Row 2 in Fig. 4b). The ideal output (Truth Table) and the experimental results are shown in Row 3 and Row 4 of Fig. 4b, respectively. All the experimental output data are consistent with the simulation results.

**Fig. 5:**
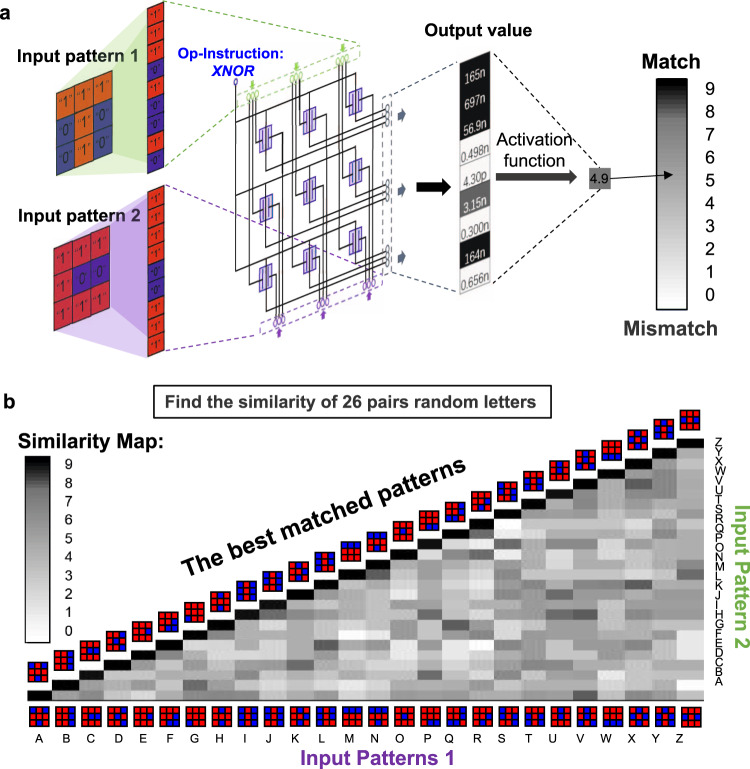
Demonstration of the image comparing function based on logic *XNOR*. **a** Diagram of the working process of image similarity function. Two 3 × 3-pixel patterns are input in parallel from each of the two input ports of the array. The current at each pixel is processed by the activation function and then summed, end up with a value between 0 and 9 to represent the similarity of the image. **b** A custom 3 × 3-pixel alphabetic A–Z patterns are shown in the horizontal axis. Using the processing method in **a**, we made a pairwise comparing of the 26 letter patterns, the activated value map is obtained. The best-matched patterns on the diagonal indicate that it realises the function of image similarity.

The demonstration of image similarity comparison is shown in Fig. 5a. The input graphs are processed and encoded in the same way as the former, and the Op-Instruction is set to 5 V (*XNOR*). To judge the similarity of two input images, the 9 × 1 output data need to be put into an activation function (sigmoid function) to obtain the final result. After the sigmoid function process, the values sum to obtain a final score (0–9). The score measures whether the two images are matching (9), mismatching (0) or kind of matching (0~9). Figure 5b shows the test data of 26 randomly compared letter patterns. Only when the two letters are the same can the system output a value close to 9, and different letters can output how similar the two letters are. More details and data processing methods are described in Supplementary Note 7.

## Discussion

In conclusion, we demonstrate a low transistor consumption image processing array based on TSC WSe_2_ transistors. Compared with existing complex logic circuits, this array system uses a single transistor to implement a pixel processing unit, greatly reducing circuit redundancy and improving transistor utilisation. Because the TSC WSe_2_ transistor can electrically switch between *AND* and *XNOR* logic functions, two different image process tasks can be carried out in the same hardware without complex multiplexer circuits. We believe that this low transistor consumption scheme has the potential to solve the circuit redundancy issue in parallel computing.

## Methods

### Fabrication of 3 × 3 pixel processing array

We mechanically exfoliated an ~60 × 90 μm^2^ WSe_2_ flake from a bulk crystal (from HQ Graphene) and transferred it onto a SiO_2_/Si (300 nm SiO_2_ grown on p-doped Si substrates) substrate. Reactive ion etching with Ar/CF_4_ plasma can be used to thinner the material if needed. Then, a 3 × 3 WSe_2_ flake array is patterned from it by defining a mask with electron-beam lithography (EBL) and reactive ion etching with Ar/CF_4_ plasma to remove the unmasked material. The Cr/Au stacks (4 nm/18 nm) were patterned and deposited on another SiO_2_/Si substrate as the bottom gate electrode by using EBL and electron-beam evaporation (EBE). Then, a 30-nm-thick Al_2_O_3_ gate oxide was deposited by atomic layer deposition (ALD) as the back gate dielectric. The patterned WSe_2_ flakes were transferred from the SiO_2_/Si substrate to the correct position of the Al_2_O_3_ gate oxide under an optical microscope by using a water-soluble transparent PVA film. Next, the source and drain electrodes were also patterned by Cr/Au stacks, and the top gate oxide (~30 nm Al_2_O_3_) was deposited on the channel surface by ALD (2 nm Al_2_O_3_ deposited by EBE as a seed layer). Finally, the top gate electrode pattern is deposited by EBE. After fabrication, the devices were annealed at 250 °C in a nitrogen atmosphere for 2 h to ensure good contact between the metal electrode and semiconductor.

### Electrical measurements

In this study, all electronic measurements were performed at room temperature and under ambient conditions. The electronic measurements were conducted using a commercial KEYSIGHT B1500A source/measure unit on a probe station (Cascade Summit 11000).

## Supplementary information

Supplementary Information

## Data Availability

Relevant data supporting the key findings of this study are available within the article and the Supplementary Information file. All raw data generated during the current study are available from the corresponding authors upon reasonable request.
