# Supplementary material for: An application-specific image processing array based on WSe2 transistors with electrically switchable logic functions
Source: Nat Commun. 2022 Jan 10;13:56. doi: 10.1038/s41467-021-27644-3 (PMC8748635; doi:10.1038/s41467-021-27644-3)
Supplement: Supplementary file 1 — Supplementary Information [file 41467_2021_27644_MOESM1_ESM.pdf]

An application-specific image processing array based on WSe_2 transistors with electrically switchable logic functions

Senfeng Zeng[1], Chunsen Liu[1, 2, *], Xiaohe Huang[1], Zhaowu Tang[1], Liwei Liu[1, 2] and Peng Zhou[1, 2,3 *]

[1]State Key Laboratory of ASIC and System, School of Microelectronics, Fudan University, Shanghai 200433, China, [*]E-mail: pengzhou@fudan.edu.cn

[2]Frontier Institute of Chip and System&Qizhi Institute, Fudan University, Shanghai 200433, China

[*]E-mail: chunsen_liu@fudan.edu.cn

[3]Shanghai Frontier Base of Intelligent Optoelectronics and Perception, Institute of Optoelectronics, Fudan University, Shanghai 200433, China

Table of content

Supplementary Note 1: Preparation method of the thin-film array.

Supplementary Note 2: Fabricating process of the array.

Supplementary Note 3: Application of the chemical vapour deposition（CVD) WSe_2 film.

Supplementary Note 4: Controllable switching performance of the devices.

Supplementary Note 5: Computing method of transistor consumption in Table 1.

Supplementary Note 6: Characterization of 3 × 3-pixel homogeneity.

Supplementary Note 7: Processing method of test data.

Supplementary Note 1: Preparation of channel films.

WSe_2 shows a strong thickness-dependent polarity. This is determined by its band structure and carrier concentration under different thicknesses. It has been shown that two-dimensional (2D) material WSe_2 has a better hole transport at about 10 layers[1]. The realization of the two functions of our array depends on the switching between bipolar and N-type of channel WSe_2. Therefore, to achieve the function of our array, we must select WSe_2 films in this thickness range as our device channel.

However, it is difficult to obtain large-area and flat WSe_2 film simply by mechanical stripping. We adopted the method of mechanical stripping and etching thinning to obtain a large-area film. The method shown in **Supplementary Figure 1** was adopted to prepare the channel array. Firstly we used PDMS to peel WSe_2 onto Si/SiO_2 substrate, then observed it under an optical microscope and selected the flat WSe_2 material. Following this, we used Ar/CF_4 plasma etching of WSe_2 to about 6-10 nm thickness, which enables the device to have good bipolar electrical characteristics[1, 2]. Finally, the channel array pattern is exposed by electron beam lithography (EBL), then used Ar/CF_4 plasma etching again to get the channel array.

In the above process, experiments were also designed to monitor the progress of etching the film to ensure that the film thickness was suitable. We first selected a flat WSe_2 film and obtained its initial thickness by atomic force microscope (AFM) scanning. The thickness of the selected film is then measured again after every 10 seconds of etching until 40 nm. After that, due to the thin WSe_2 thickness, we measured the thickness every 5 s after etching until the thickness was about 10 nm (12 nm). The

thickness-time curve and the microphotographs of the AFM are shown in **Supplementary Figure 2**. The thickness changes with etching time remain roughly linear which proves the feasibility of the etching process.

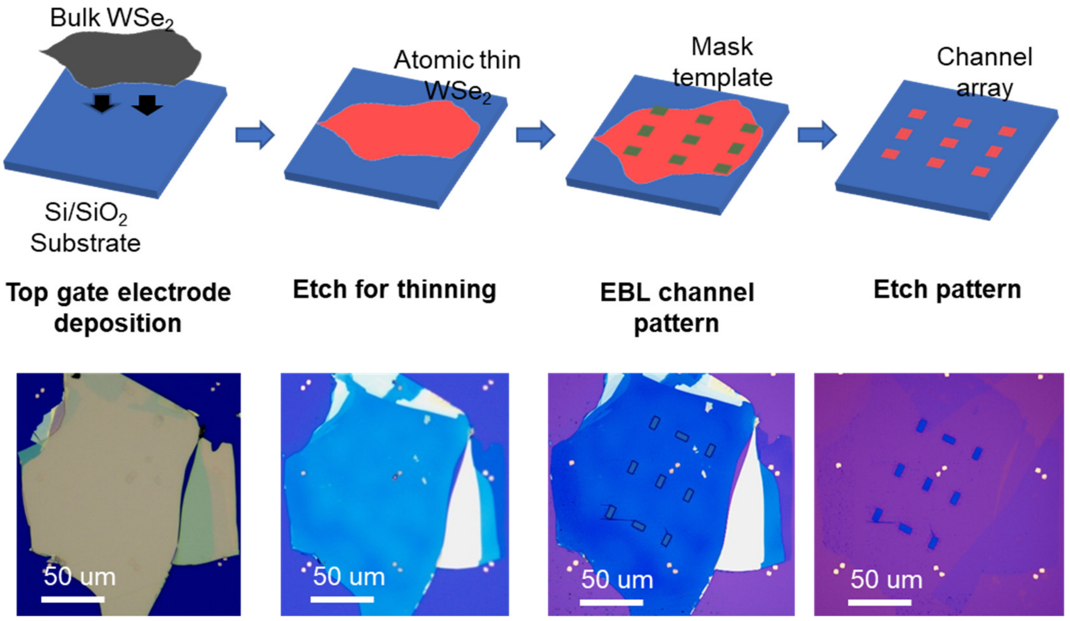

Supplementary Figure 1| Preparation method of the thin-film array. Schematic and optical photographs are displayed on the figures. The first step is to select a flat bulk material, we used PMMA to peel the material from the bulk and then directly paste it onto the Si /SiO_2 substrate surface. It is then etched using RIE. The etching for thinning step is repeated several times until we get the desired WSe_2 thickness. Then we use EBL to draw the mask pattern. Finally, the excess material is etched.

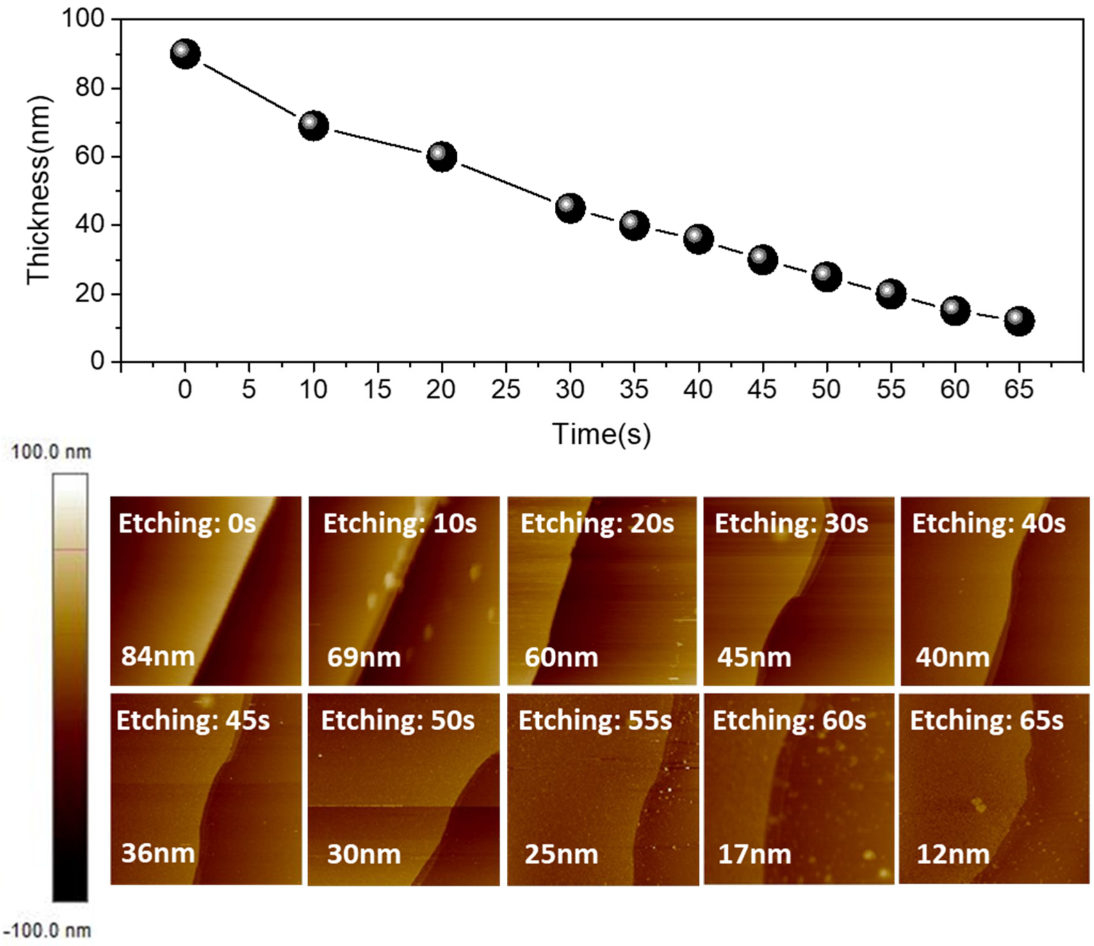

Supplementary Figure 2| Etching and thinning process of WSe_2 thin film. The thickness-time curve and the microphotographs of the AFM are caught. The thickness changes with etching time remain roughly linear.

Supplementary Note 2: Fabricating process of the array.

Here we show our device fabrication process in detail. The entire array of electrodes requires three exposures and a deposition process. We pre-designed the mask layout for each exposure processing and used the same markers to align the exposure pattern.

The preparation of the array was performed in the following seven steps as shown in **Supplementary Figure 3** and the optical image of the fabrication processing are

shown in **Supplementary Figure 4**:

- *Bottom gate electrode deposition.* The electrode pattern was exposed for the first time, and Cr/Au stacking of 4/18 nm was deposited by EBE.
- *ALD bottom gate dielectric.* The bottom-gate dielectric was then deposited by ALD. 30 nm Al_2O_3 was deposited. Trimethylaluminum (TMA) and water are used as ALD precursors.
- *Channel array transfer.* The WSe_2 channels array is etched in advance according to the electrode pattern, this process will be mentioned in section **S2**. We used PMMA + PVA to peel the etched material from the substrate and then affixed it to the appropriate position of the electrode under an optical microscope, and then soaked the substrate in deionized water for two hours to remove the PVA.
- *S/D electrode deposition.* The electrode pattern was exposed for the second time, and Cr/Au stacking of 4/18 nm was deposited by EBE.
- *Seed layer deposition.* Because there is no suspension bond on the surface of the 2D WSe_2, it is difficult to provide the adsorption point to the precursor on the surface of the channel[3, 4]. Therefore, 2 nm Al_2O_3 deposited by EBE performed as the seed layer.
- *ALD top gate dielectric.* After the deposition of the seed layer, we deposited 30 nm Al_2O_3 as the top-gate dielectric by ALD.
- *Top gate electrode deposition.* The electrode pattern was exposed for the third time, and Cr/Au stacking of 4/18 nm was deposited by EBE.
- *Annealing.* Finally, the devices were annealed at 250 °C in a nitrogen atmosphere

for 2 hours to ensure good contacts between flakes and between the metal electrode and semiconductor.

Finally, **Supplementary Figure 5** shows the high-resolution energy dispersive spectroscopy (EDS) element mapping characterizations of the device, which implies a clean heterostructure interface.

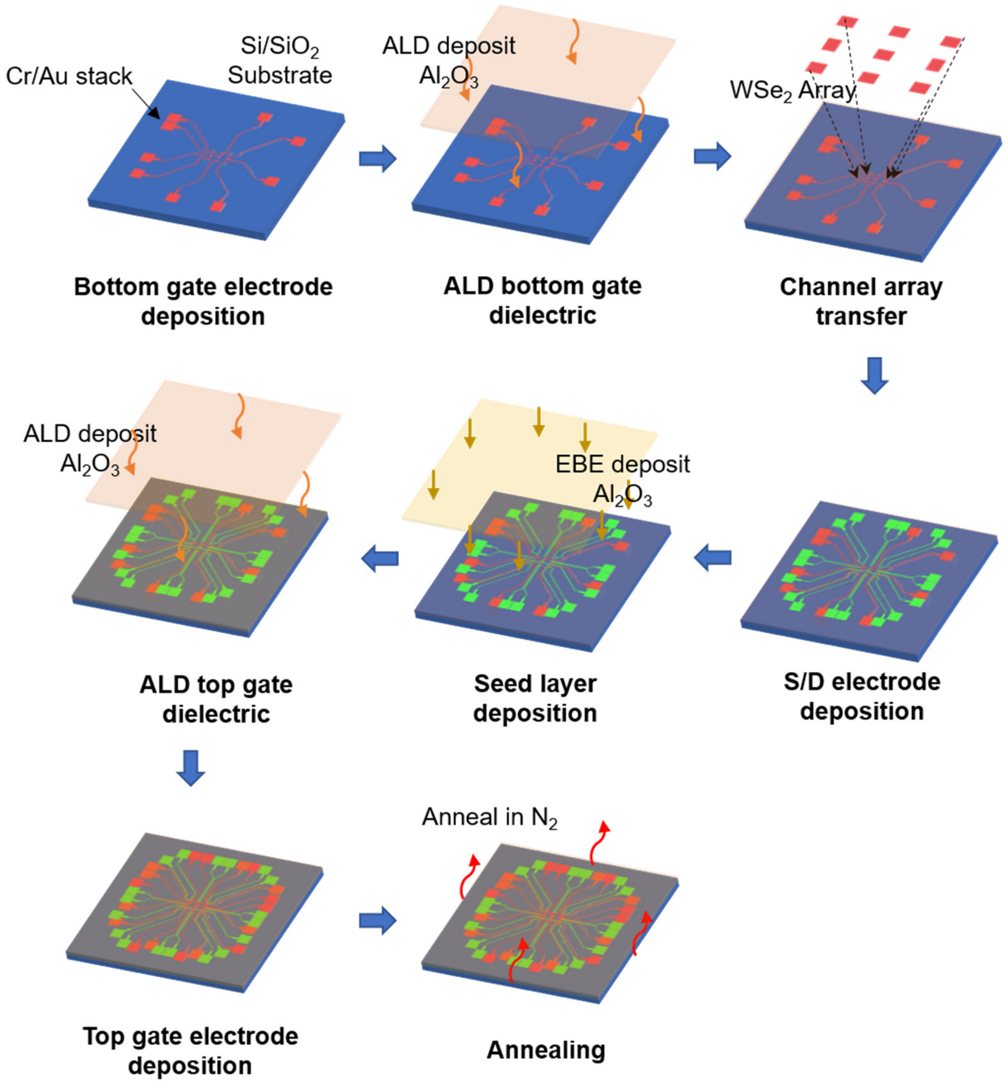

Supplementary Figure 3| The detailed fabrication process.

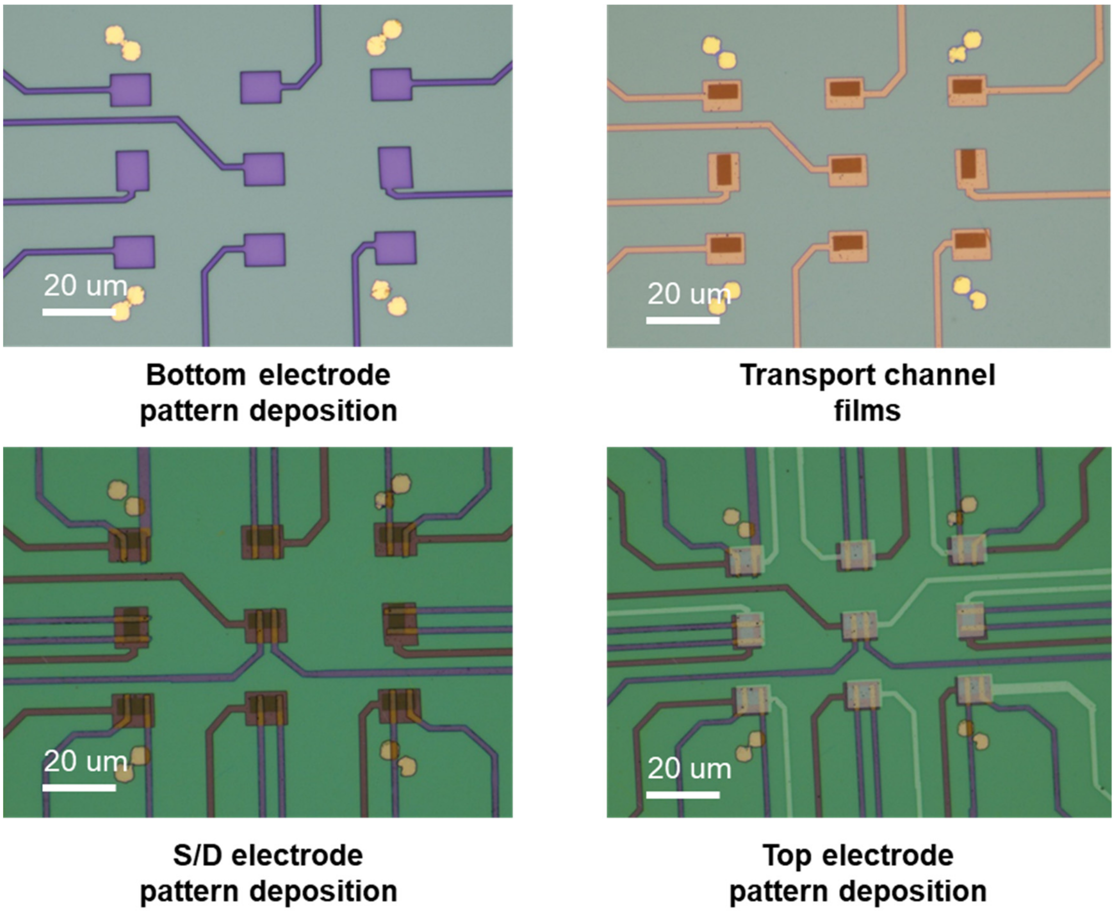

Supplementary Figure 4| The optical image of the pixel processing array. The first image shows the image after the first exposure. The nine electrodes are served as bottom input ports. The second image shows the transferred channel layer. The Al_2O_3 dielectric was deposited before transfer. Op-Instruction and output ports are then deposited as the third figure shown, after this process, we grow the top Al_2O_3 dielectric. Finally, the TSC image processing array was completed after the top input ports are deposited as the fourth image shown.

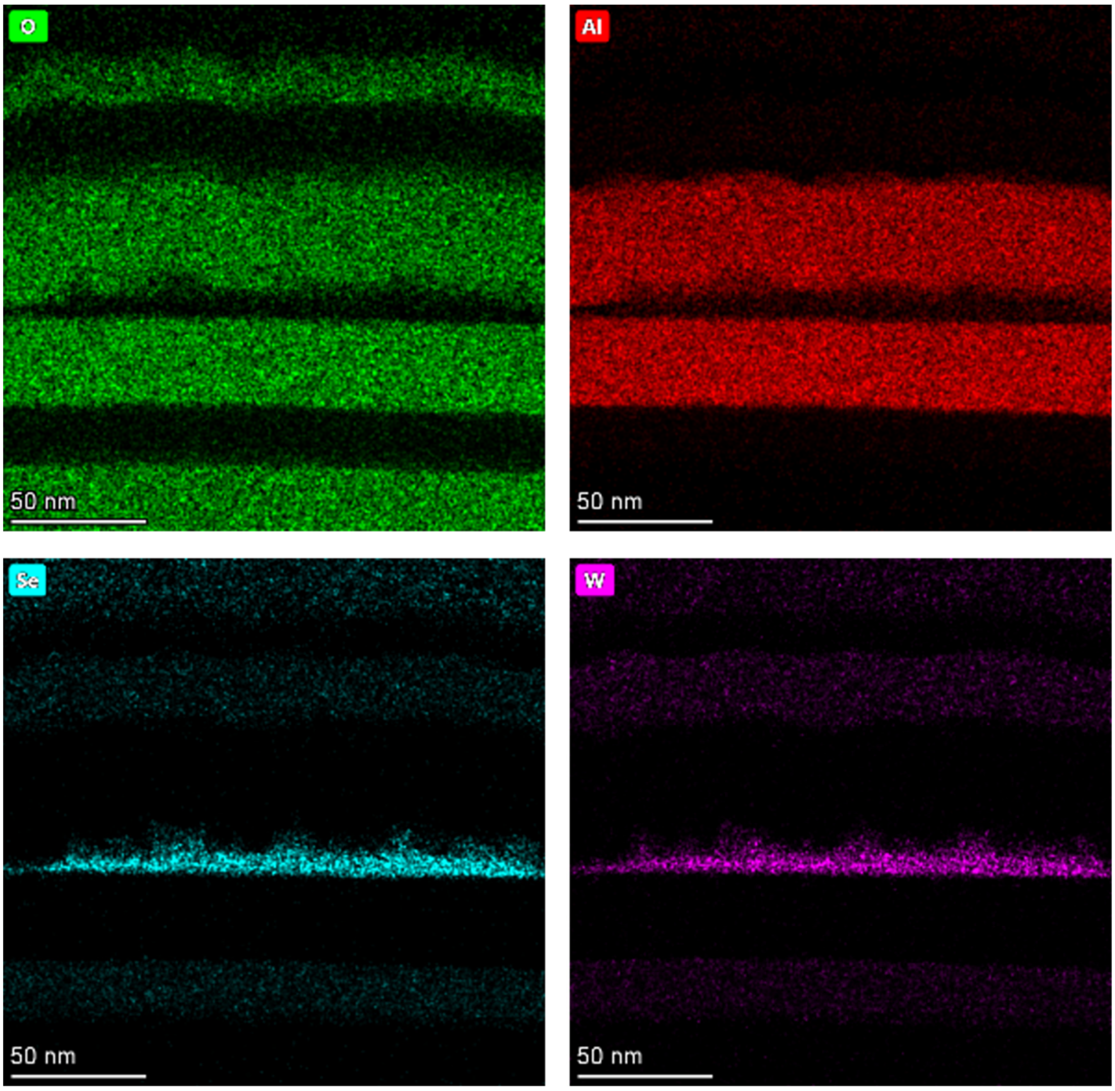

Supplementary Figure 5| Device structure and EDS characterization. The distribution of O, Al, Se and W is shown in the figures, which demonstrates clean interfaces. The EDS mapping scales are 50 nm.

Supplementary Note 3: Application of the chemical vapour deposition（CVD) WSe_2 film.

How to use 2D material film on a large scale is a big problem in the application of 2D materials. Because the CVD film has the advantage of large area and good uniformity, it is one of the most reliable methods to construct the circuit by using 2D films grown

by CVD. Here, we have also demonstrated experimentally that our process has the potential for large-scale application by using the large area CVD WSe_2 film.

The left part of **Supplementary Figure 6** is the flow chart of the array preparation. To facilitate subsequent exposure, we first transferred the CVD WSe_2 film to our marked substrate with thermal release tape. **Insets a** is a photograph of the film under the original substrate, and **Inset b** to **d** show the process of patterning to metallization. A 3×3-pixel processing array is finally successfully prepared by using the process described above, which shows that our process has good compatibility for the film of large-area CVD. As shown in the right inset, the transfer curves under different V_{DS} are also tested. The devices exhibit strong N-type characteristics, which is due to the thin thickness of the CVD WSe_2 film. In the end, the CVD WSe_2 film was not used for the actual array function test, but the feasibility of large-scale expansion was proved in the process.

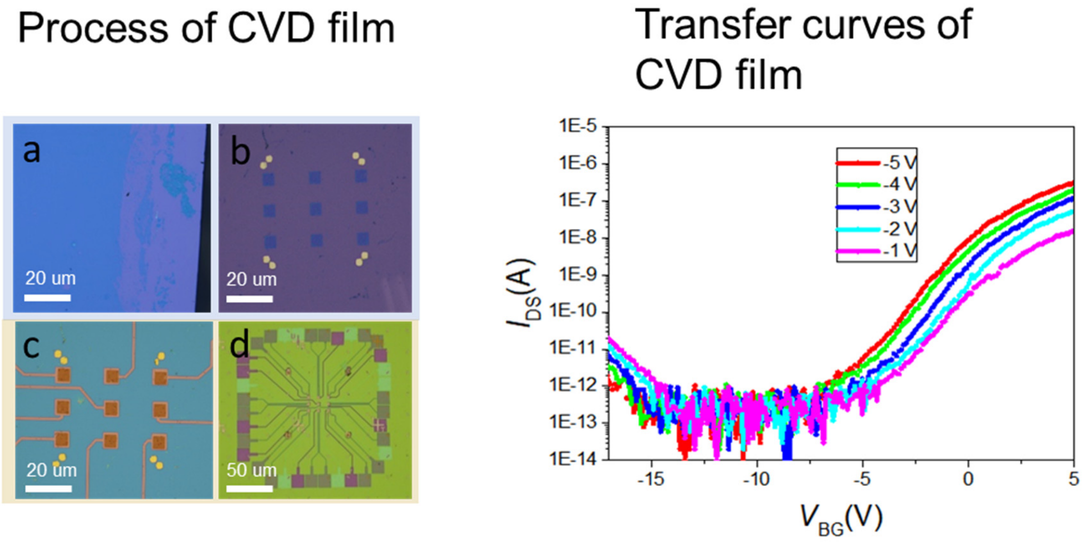

Supplementary Figure 6| The preparation process of the TSC array using CVD WSe_2 film. Left part: **a,** the optical photograph of WSe_2 on unmarked sapphire substrate.

b-c, transferring and patterning of large-area thin films. **d,** the final morphology of the TSC array. Right part: the transfer curves of the device under different V_{DS}. The threshold voltage drifts to the right V_{DS} from -5 V to -1 V, which is also in line with the mechanism explanation in our text.

Supplementary Note 4: Controllable switching performance of the devices.

In the course of our experiments, we found that the devices also have other practical properties. In a field-effect transistor, the on-off ratio measures the ability of the gate pressure to control the conduction channel. This value can be adjusted by an applied electric field. We apply an input voltage V_{CG} to the Si substrate as a control gate and then scan the back gate V_{BG} using the same test method in **Fig. 2**.

The transfer curves under different V_{CG} biases are shown in **Supplementary Figure 7**. In the process of scanning, the off-state current of the transfer curve showed two trends. When the V_{CG} is less than -4 V, the off-state current decreases with the increase of the V_{CG}; when the V_{CG} is greater than -4 V, the off-state current increases. As shown in the figure, the presence of V_{CG} causes the off-state current of the transistor device to vary by several orders of magnitude, which is enough to improve the logic performance of the device. We hypothesize that this regulation is related to the existence of the buried metal electrode. When there is no buried gate electrode, the V_{CG} has uniform control over the whole channel. Due to the existence of the buried metal gate, the control of the channel by V_{CG} is shielded. The electric field introduced by the V_{CG} can only be applied near the source and drain. So we speculate that the reason for this

phenomenon is that the introduction of V_{CG} changes the Schottky barrier between the source/drain metal and the channel. We have drawn an I_{DS}-V_{BG}/V_{CG} map where the switching ratio reaches a maximum of over 10^5 when V_{CG} = -4 V. We do not go into depth here because the on/off ratio is sufficient for us to test when the V_{CG} is 0 V.

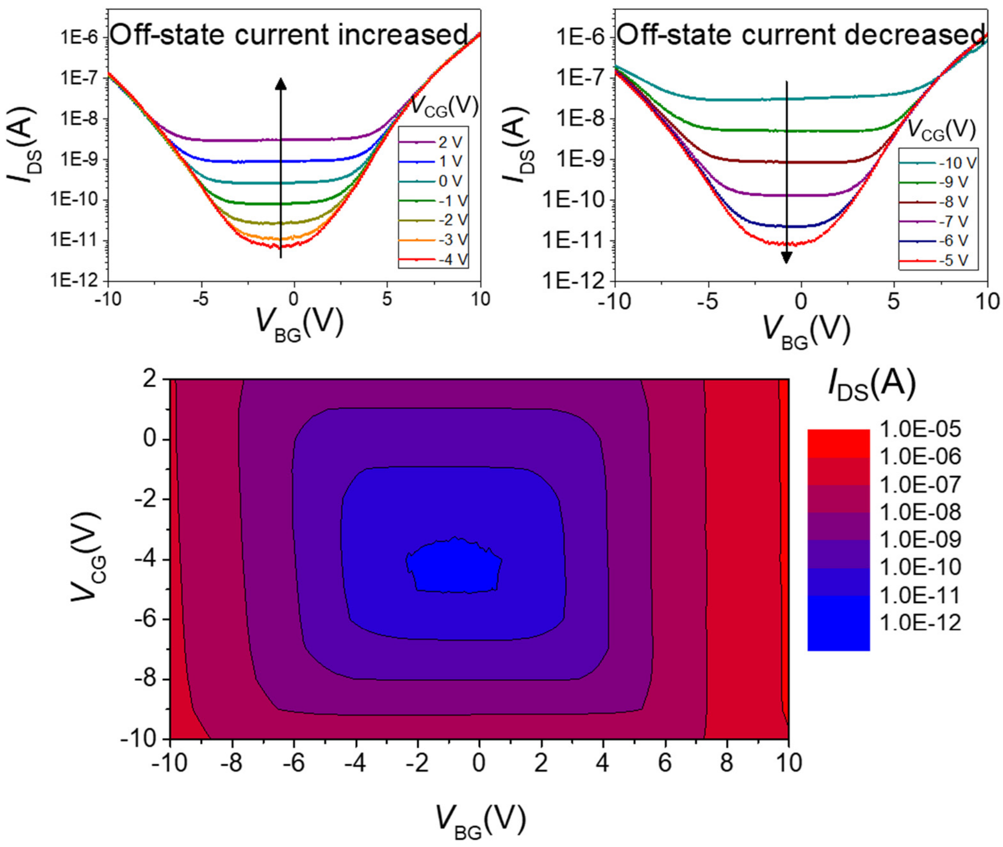

Supplementary Figure 7| Switching on/off ratio that can be modulated by V_{CG}. The V_{CG} is applied on Si substrate and under the buried gate as a control gate. The I_{DS}-V_{BG} curves show good bipolarity of the WSe_2 channel. We applied different V_{CG}, and the off-state current increased from -4V to 2V, decreased from -10V to -5V. The current colour map was drawn to see the on/off ratio more clearly. The highest on/off ratio can be seen at V_{CG} = -4V.

Supplementary Note 5: Computing method of transistor consumption in Table 1.

In **Table 1**, we set out the calculation method of transistor consumption of various emerging logic circuits. Here, we first give the gate circuit diagrams of logic *NAND*, *NOR,* and *XOR*-based on silicon NMOS logic as shown in **Supplementary Figure 8**, and using the number of transistors they need as the transistor consumption baseline of various logic gates (9 for *XOR*, 3 for *NAND*, 3 for *NOR*). We use the following formula to evaluate the complexity of each work in **Table 1**,

$$Transistor\ \ consumption = \frac{T_{XOR}+T_{NAND}+T_{NOR}\ \ (based\ \ on\ \ emerging\ \ logic)}{T_{XOR}+T_{NAND}+T_{NOR}\ \ (based\ \ on\ \ NMOS\ \ logic)} \times PG \qquad (1)$$

where T_{XOR}, T_{NAND}, T_{NOR} indicates the number of transistors required to implement logic *XOR*, *NAND,* and *NOR*, respectively. PG represents the number of planar gates in a single transistor.

For all the reported works based on depletion-load NMOS[5, 6, 7, 8], the transistor number is 9 for *XOR*, 3 for *NAND*, 3 for *NOR* (the transistor consumption is 1). It's should be noted that some of these works have not adopted the optimal circuits design, the transistor consumptions are larger than the baseline. But once they use the optimal circuits the transistor consumption will decrease to the baseline value.

The reconfigurable logic uses the same circuits to implement different logic functions, which can reduce the transistor consumption of the logic circuit. As shown in the top column of **Supplementary Figure 9**, one approach is to use an additional gate to control the polarity of the transistor. References 9-10 use the changes in polarity

to achieve the reconfiguration of the logic function (4 transistors for *NAND&NOR*, 5 for *XOR*)[9, 10]. Reference 11 realized a simpler reconfigurable logic gate by redesigning the circuit[11], logic *AND* (*NAND*) and *OR* (*NOR*) can be switched with only two transistors (2 transistors for *NAND&NOR*). In this approach, each device has two gates in the same plane, and the planar gate number of the transistor should be considered in the transistor consumption computing process. Therefore, the transistor consumption is 1.2 and 0.66 for references 9-10 and 11, respectively. The bottom column of **Supplementary Figure 9** shows another approach, the same logic circuit has a different logic function by utilizing the memory states of the device. Reference 12 uses 9 transistors to successfully implement both *NAND&XOR*[12]. When the floating transistors are semiconducting state, logic *XOR* is implemented; When they are insulating state, logic *NAND* is achieved. The original circuit in this paper is shown on the left. However, two inverters (used to convert the A, B to $\bar{A}$, $\bar{B}$) are omitted and we have supplemented its circuit as the right section. The transistor consumption of reference 12 is 0.75.

As shown in **Supplementary Figure 10**, the TSC transistor also shows the switchable logic, a single transistor sufficient for logic function. Reference 13 has used different polarity channel materials to implement a single transistor logic gate[13]. The transistor number is 2 for *NAND*, 2 for *NOR*, 2 for *XOR*. The transistor consumption is 0.4. Reference 14 shows the switchable logic of TSC transistor based on optical control signals, logic *NAND* and *NOR* are achieved in light and dark environments, respectively (2 transistors for *NAND&NOR*)[14]. The transistor consumption is 0.33.

Supplementary Table 1 has summarized the logic circuits of various emerging transistor technology and the computing process of their transistor consumption.

The results show that our device has an obvious advantage in the complexity of the logic circuit. Compared with the traditional logic circuit based on NMOS, the transistor consumption of our pixel processing unit is only 0.16 (1 transistor for *AND&XNOR* (current output), 2 transistors for *NAND&XOR* (voltage output)), which is reduced by 84%.

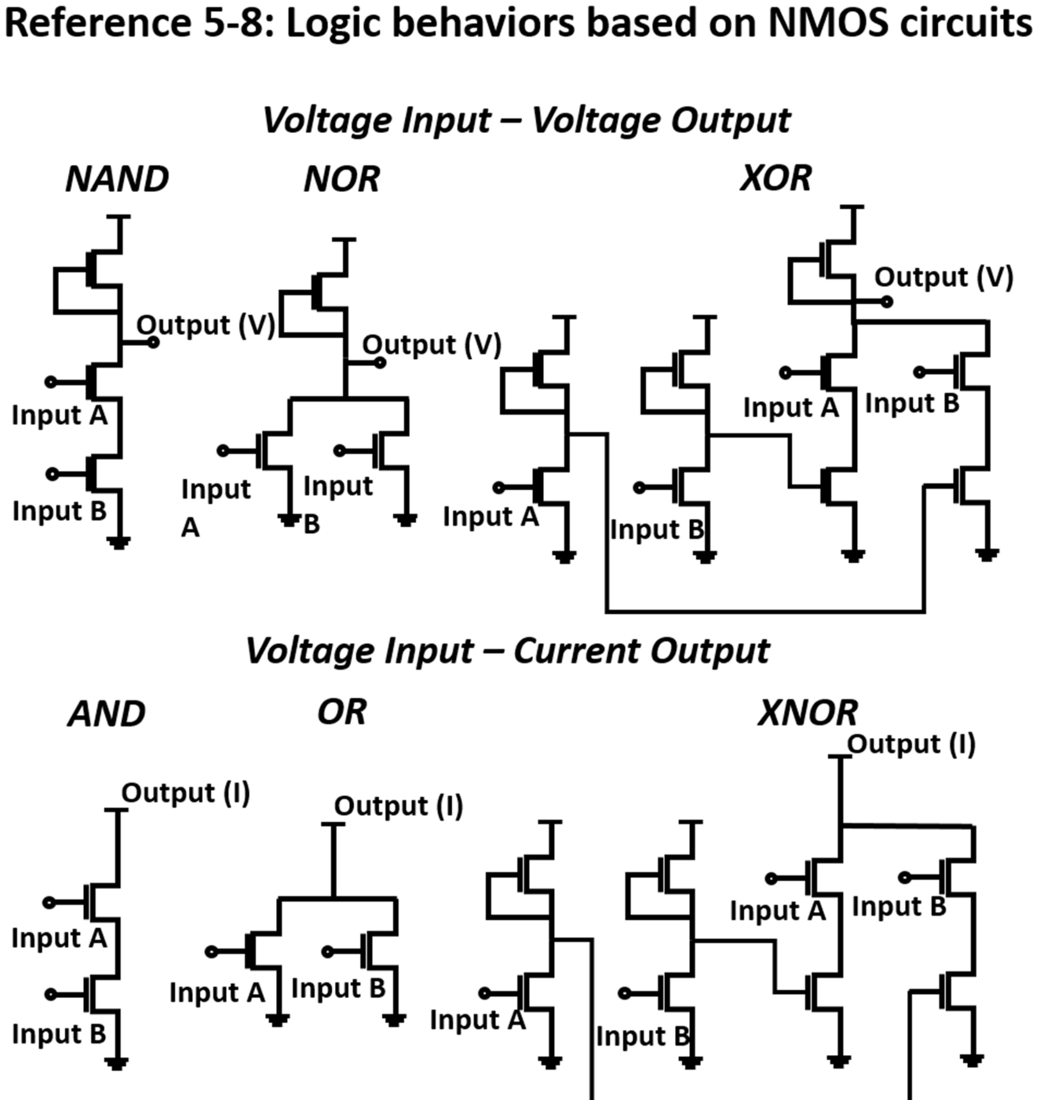

Supplementary Figure 8| The logic gate circuits based on NMOS. There are two

types of circuits, depending on whether the output signal is current or voltage. When voltage is the output signal, logic *NAND*, *NOR*, and *XOR* are equivalent to logic *AND*, *OR*, and *XNOR* when current is the output signal.

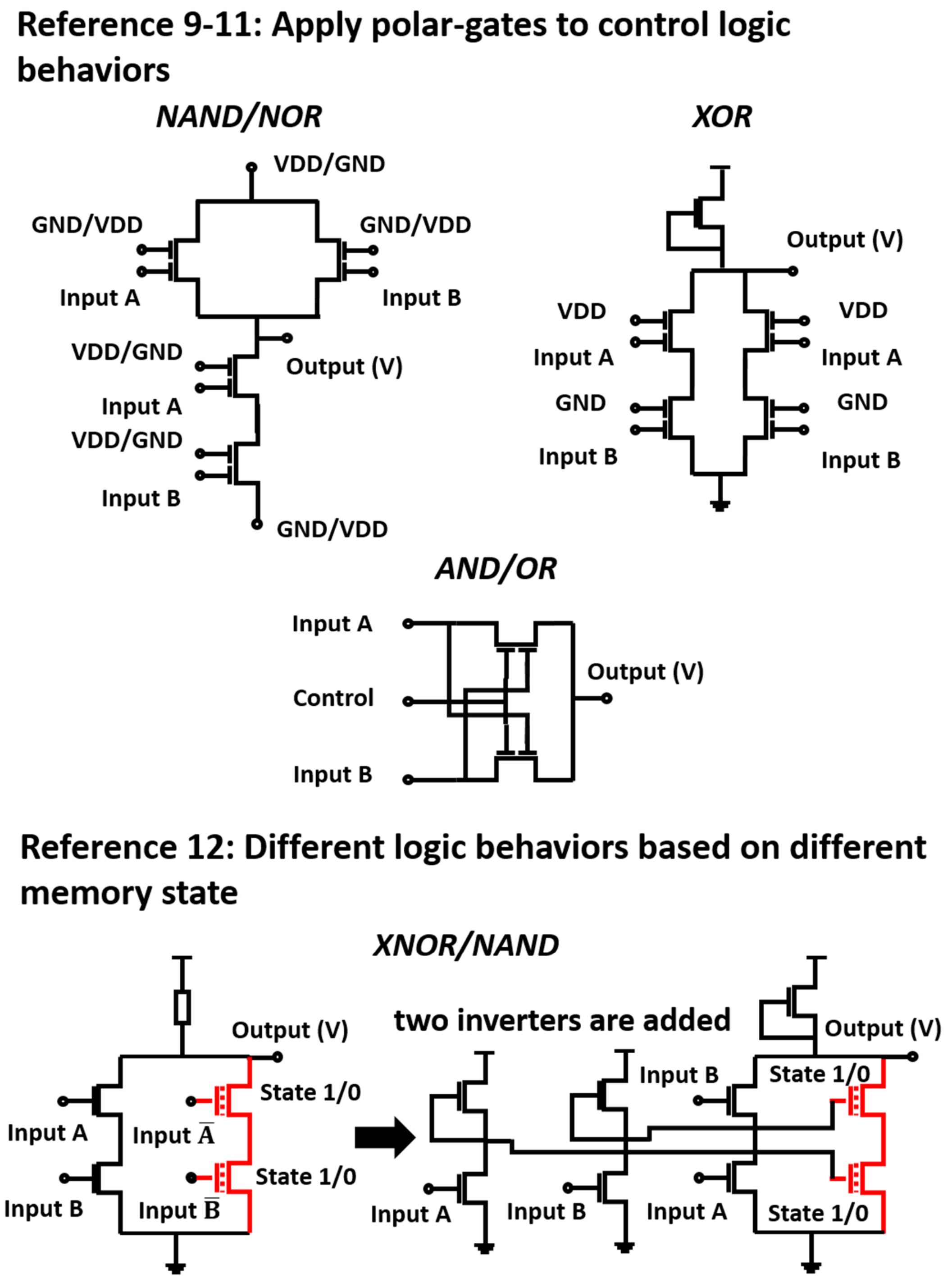

Supplementary Figure 9| Switchable logic function based on polar gate and memory state. top row: logic gate circuits based on polar-gates control. Additional gate

controls the polarity of the device, so different logic functions can be implemented in the same circuits. bottom row: the switchable logic gate based on memory state control. state 1: the channel state of red devices are insulators, logic *NAND* is implemented. state 0: the channel state of red transistors are semiconductors, logic *XOR* is implemented.

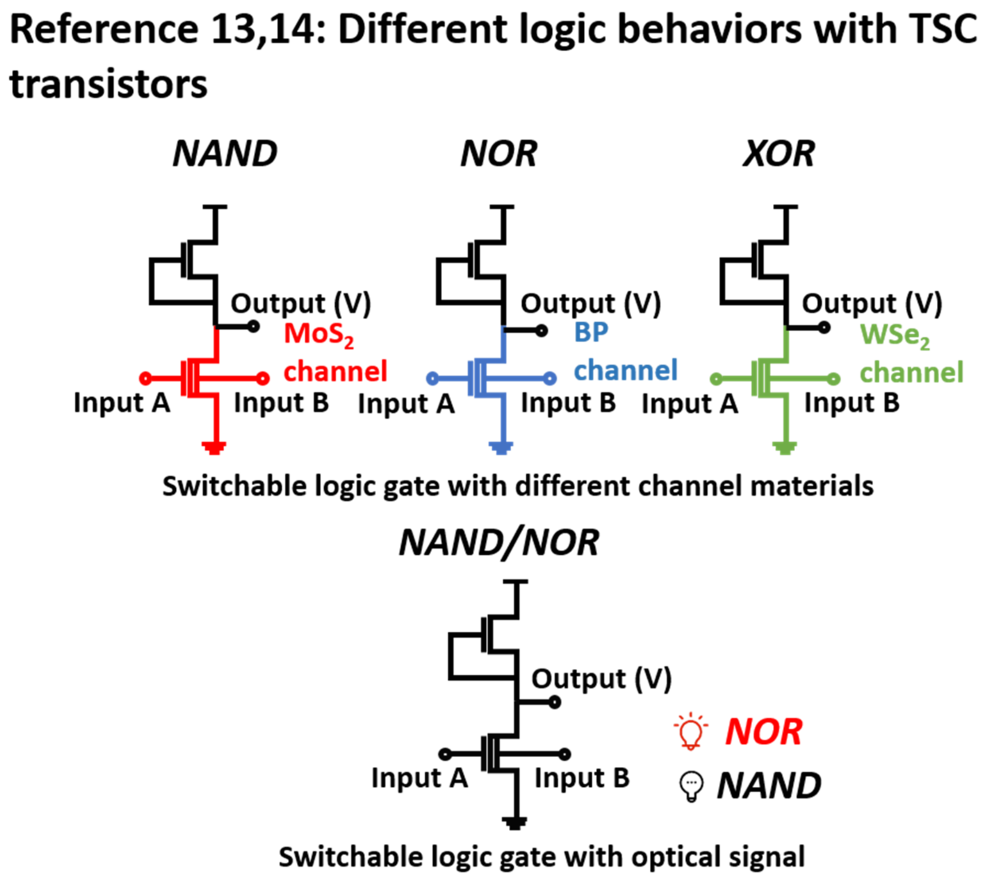

Supplementary Figure 10| Different logic gate circuits with TSC transistors. Top row: Logic function switchable TSC transistors based on different channel materials (MoS_2 for *NAND*, BP for *NOR*, WSe_2 for *XOR*). By applying different channel materials, different logic functions are realized. Bottom row: Logic function switchable TSC transistor based on optical signal (light for *NOR*, dark for *NAND*).

Supplementary Table 1| The circuits diagram and the corresponding transistor consumption of the emerging logic gate work.

Reference	Circuit diagram	Logic Function	Transistor number	Transistor consumption
5-8	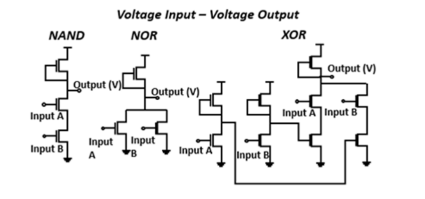	*NAND*	3	$\frac{9(XOR)+3(NAND)+3(NOR)}{9(XOR)+3(NAND)+3(NOR)} \times 1$ = 1.0
		NOR	3	
		XOR	9	
9,10	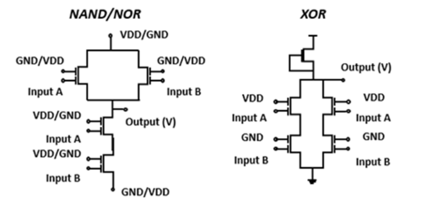	*NAND & NOR*	4	$\frac{5(XOR)+4(NAND\&NOR)}{9(XOR)+3(NAND)+3(NOR)} \times 2$ = 1.2
		XOR	5	
11	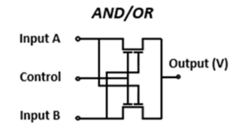	*(NAND & NOR)*	2	$\frac{2(NAND\&NOR)}{3(NAND)+3(NOR)} \times 2$ = 0.66
12	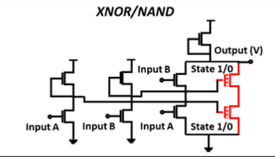	*NAND & XNOR*	9	$\frac{9(XOR\&NAND)}{9(XOR)+3(NAND)} \times 1$ = 0.75
13	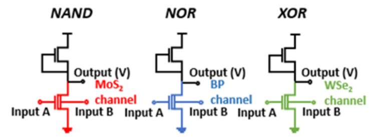	*NAND*	2	$\frac{2(XOR)+2(NAND)+2(NOR)}{9(XOR)+3(NAND)+3(NOR)} \times 1$ = 0.40
		NOR	2	
		XOR	2	
14	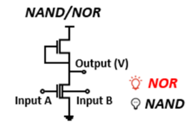	*NAND & NOR*	2	$\frac{2(NAND\&NOR)}{3(NAND)+3(NOR)} \times 1$ = 0.33

Supplementary Note 6: Characterization of 3 × 3-pixel homogeneity.

In **Fig. 3**, we extracted the test data of the first pixel in the array to demonstrate the switchable logic function of the TSC pixel processing array. It can be seen that the logic function of a single device is switched from *AND* to *XNOR* as the Op-Instruction

voltage changes. In image data processing, the pixels of the image are parallelly processed by the TSC transistor array, so the output value of the devices must be uniform.

To evaluate the device homogeneity and reliability of the logic behaviour, we present raw test data for the logical output current of 9 pixels in our array as a supplement to **Fig. 3**. As shown in **Supplementary Figure 11**, all nine devices output a steady logical current over the operating voltage range. To ensure that the output logic is correctly displayed as *AND* & *XNOR*, it is important to ensure that all output currents have a “boundary” between “0” and “1” over all devices. Because different V_{DS} voltages are used, the "0" state current values under logic *AND* & *XNOR* are different to some extent. The “boundary” for logic *AND* is 100 *pA*. In logic *XNOR*, the “boundary” is 2 *nA*. **Supplementary Figure 12** visually shows the boundary and output current under the two different logic functions. The lower-left corner and upper-right corner of each map represent the logical output current under the hole and electron conduction, respectively. Based on this output current characteristic, it is demonstrated that the device can uniformly switch from the logic *AND* function to the logic *XNOR*. The homogeneity of the pixel array is guaranteed.

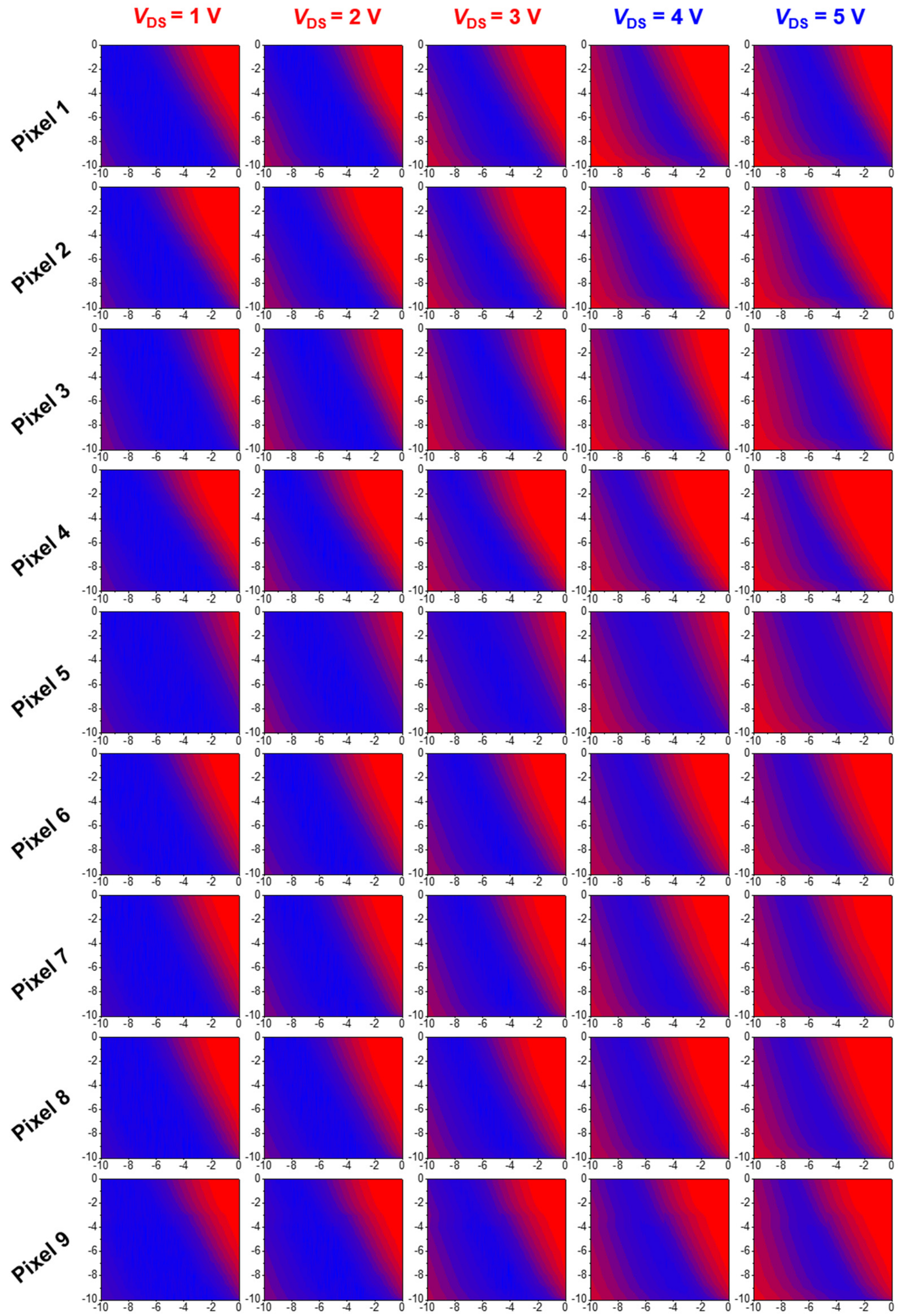

Supplementary Figure 11 | The colour map of I_{DS} under the control of V_{TG} & V_{BG} at different V_{DS} from 1 V to 5 V. The horizontal and vertical coordinates of each map are the same as in **Figure. 3**. During the V_{DS} increase from 1 V to 5 V, the logic changes of the nine devices are the same as those of the individual devices in **Fig. 3**, and the

switching current values of the different devices are kept within an acceptable error range.

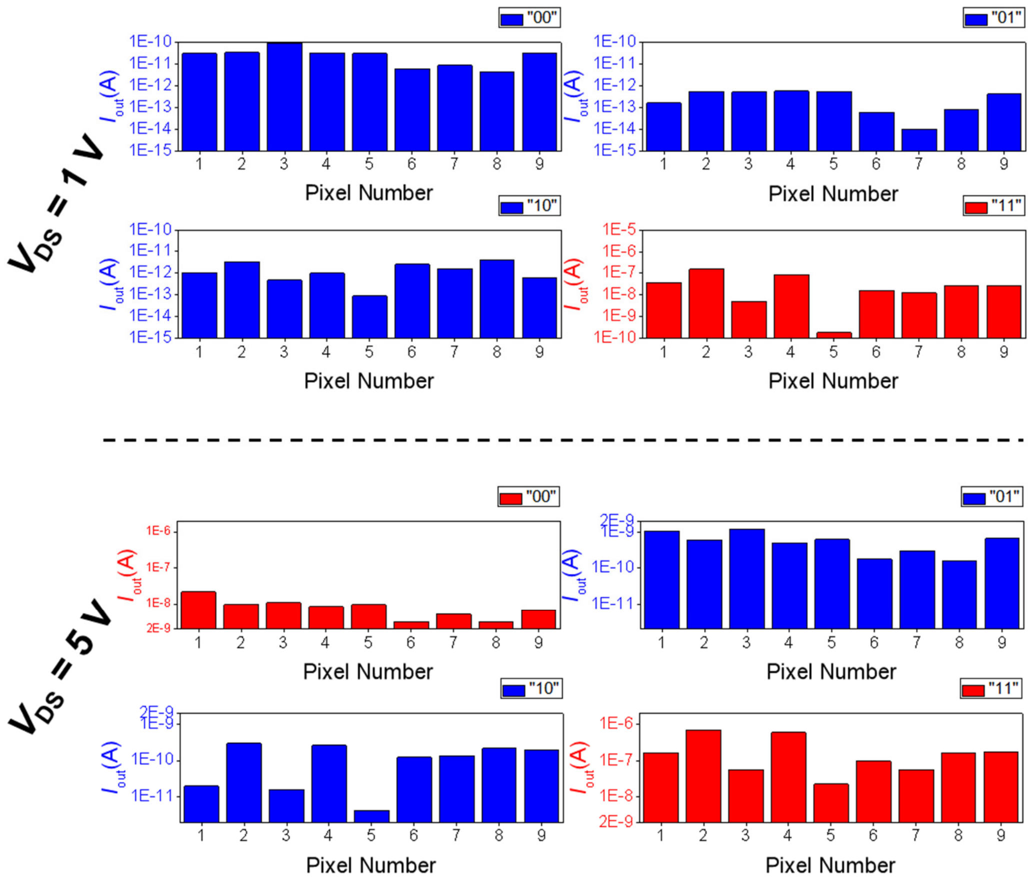

Supplementary Figure 12| The output current of the devices under different logic inputs.

Supplementary Note 7: Processing method of test data.

The process flow of the TSC pixel processing array is shown in **Supplementary Figure 13**. Two input matrices are delivered by the two 3 × 3 setting ports. The two computing methods of *AND* & *XNOR* are determined by the voltage level of the Op-Instruction. The functions of finding the intersection and similarity of images are performed by

AND & *XNOR* computing respectively.

Supplementary Figure 14 restores the original form of the 100 pairs of data in **Fig. 4**. The 100 pairs of flattened random images are unfolded into 3 × 3-pixels here to give a more intuitive view of the image intersection function. In **Supplementary Figure 15**, we also completely demonstrate our method of data processing in *XNOR* computing. In the output matrix array, the darker the colour, the higher the output current value. The matrix on the diagonal, where all nine pixels are dark, represents the two inputs are exactly similar. After calculating the two groups of inputs, we use the activation function $F(\mathrm{x}) = \mathrm{Sigmoid}\,(-10\ \times \mathrm{lg}(\frac{x}{50\ nA}))$ to process the output current value (x represents the output current I_{DS} of each pixel, and the Sigmoid function is defined by the following formula: $\mathrm{Sigmoid}(x) = \frac{1}{1+e^{-x}}$), then the final output can be seen in **Fig. 5c**.

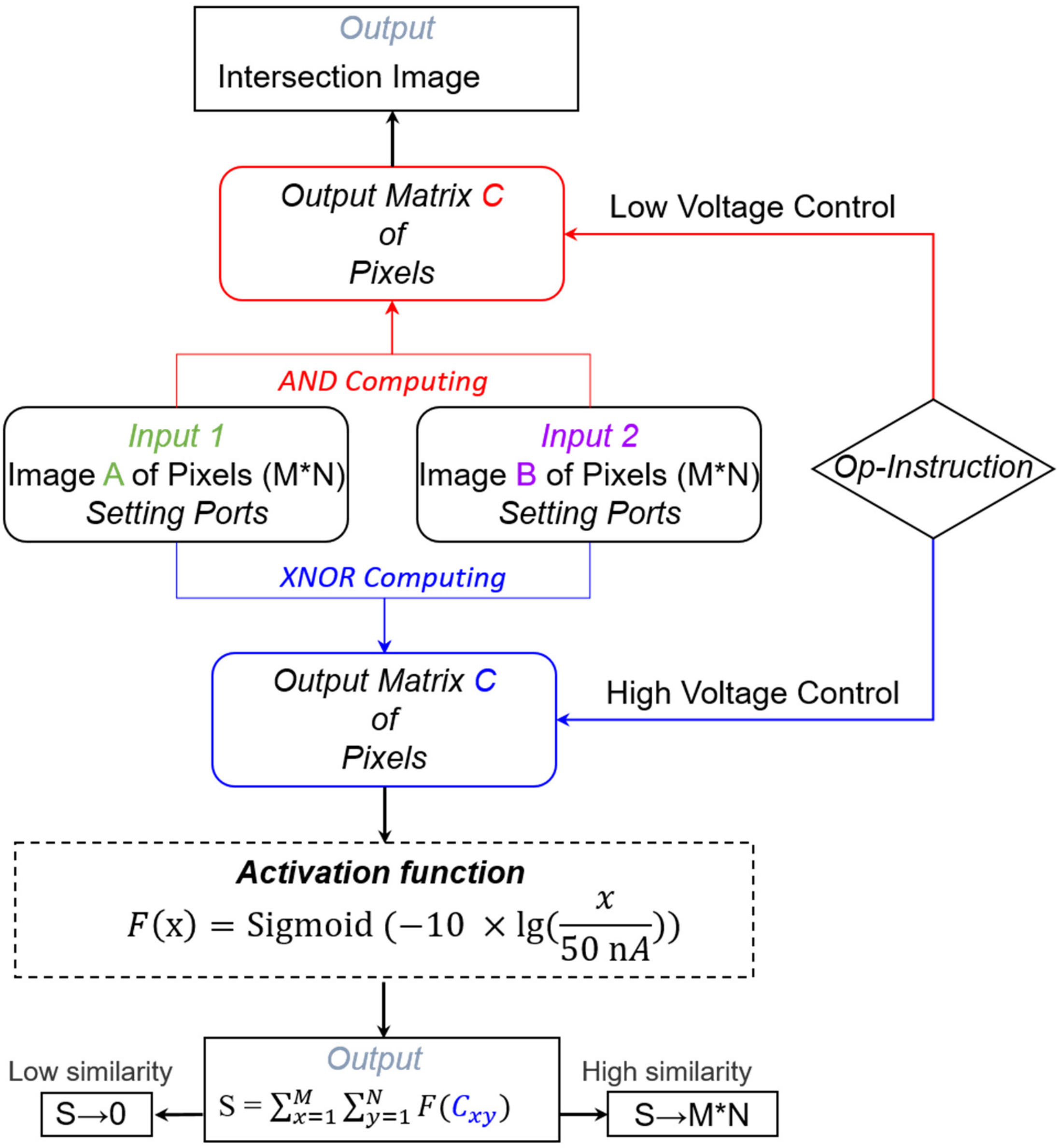

Supplementary Figure 13| The workflow chart of the pixel processing array. The data processing process begins in the middle of the chart, with two 3×3-pixel data input from two strings of input ports in the array. At the same time, the functions that the array needs to perform are determined based on the voltage of the Op-Instruction ports. For the function of finding image intersection, the calculated results are directly output as 3×3-pixel data of equal rank. For the function of finding image similarity, the output data is processed by our activation function for summation, and the judgment is made

according to the summation result.

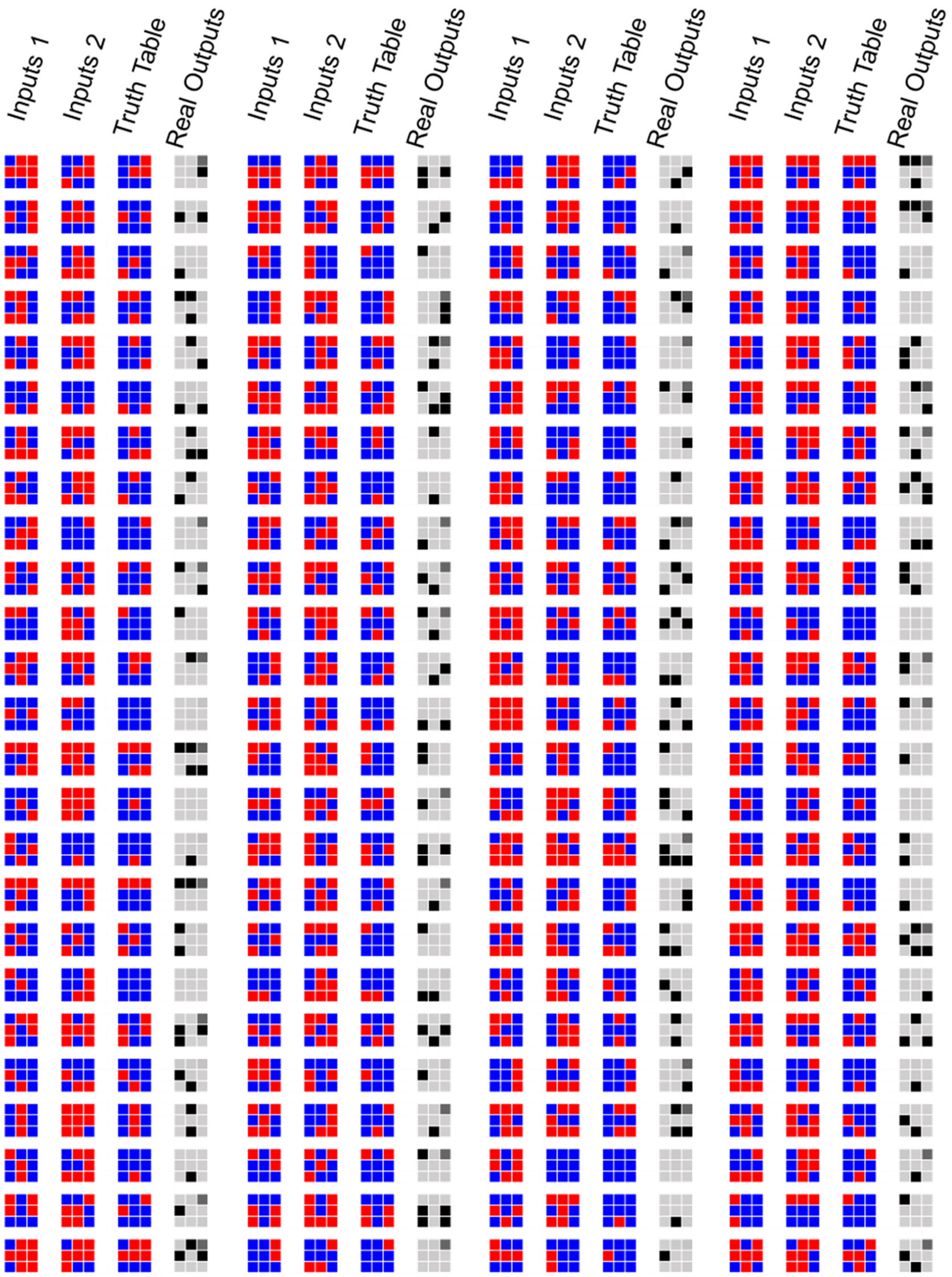

Supplementary Figure 14 | The random 100 pairs of image intersections in Fig. 4. The Flattened data in **Fig. 4** is restored here. Input data, truth tables, and actual output can be compared more intuitively. The processing and colour bars of the data here are the same as in **Fig. 4**.

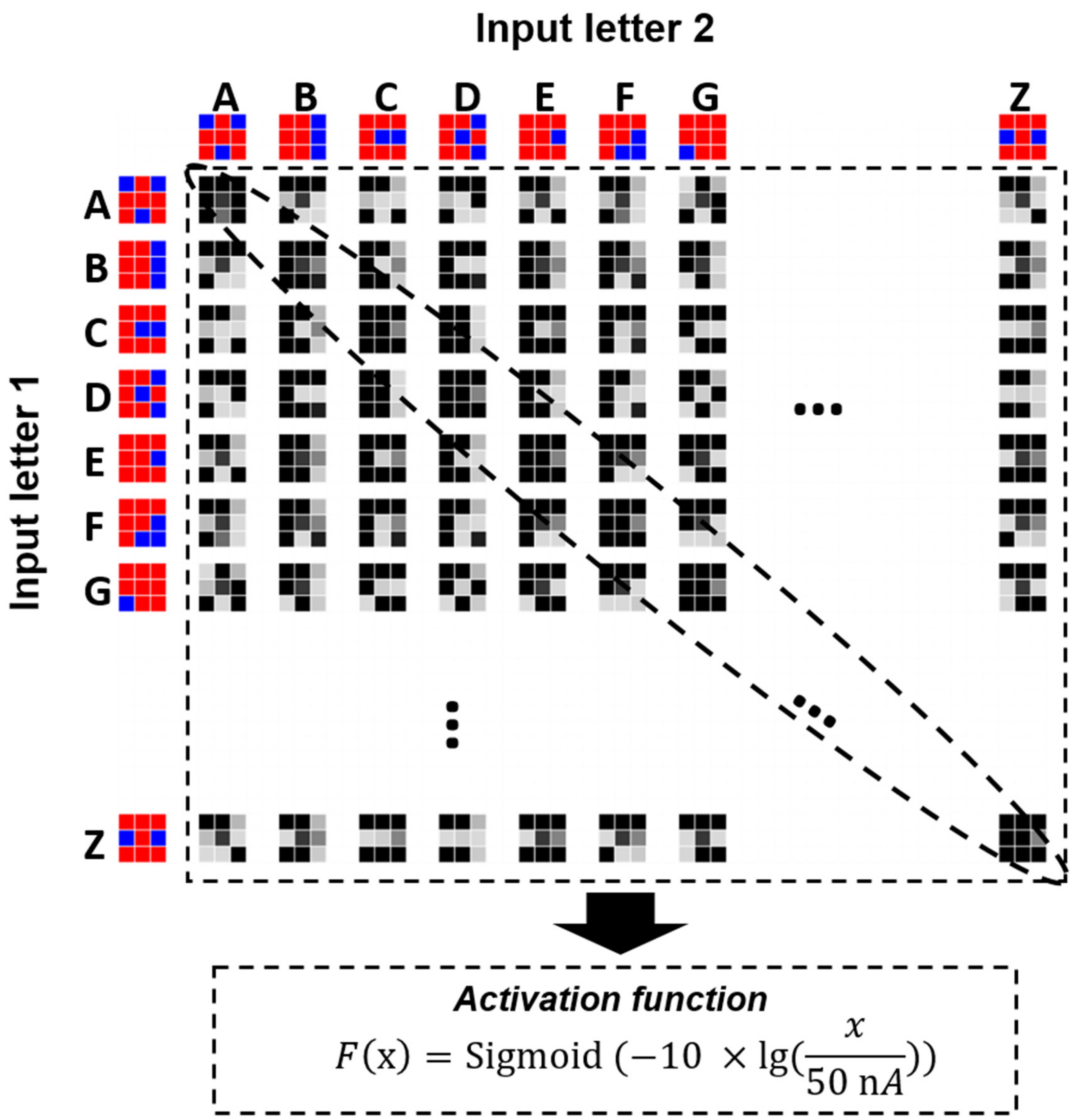

Supplementary Figure 15| The data processing method of Fig. 5. By pairwise comparing a custom set of letters, we can get a 26 × 26 scale array. Activation functions are applied to each 3 × 3 pixel array to obtain the similarity map shown in **Fig. 5c**.

Reference

1. Wang Z*, et al.* The ambipolar transport behavior of WSe2 transistors and its analogue circuits. *NPG Asia Mater* **10**, 703-712 (2018).
2. Das S, Appenzeller J. WSe2 field effect transistors with enhanced ambipolar characteristics. *Appl Phys Lett* **103**, 103501 (2013).
3. Liu H, Xu K, Zhang X, Ye PD. The integration of high-k dielectric on two-dimensional crystals by atomic layer deposition. *Appl Phys Lett* **100**, 152115 (2012).
4. Radisavljevic B, Radenovic A, Brivio J, Giacometti V, Kis A. Single-layer MoS2 transistors. *Nat Nanotechnol* **6**, 147-150 (2011).
5. Lin Z*, et al.* Solution-processable 2D semiconductors for high-performance large-area electronics. *Nature* **562**, 254-258 (2018).
6. Wachter S, Polyushkin DK, Bethge O, Mueller T. A microprocessor based on a two-dimensional semiconductor. *Nat Commun* **8**, 14948 (2017).
7. Yu L*, et al.* Design, Modeling, and Fabrication of Chemical Vapor Deposition Grown MoS2 Circuits with E-Mode FETs for Large-Area Electronics. *Nano Lett* **16**, 6349-6356 (2016).
8. Xiang L*, et al.* Low-power carbon nanotube-based integrated circuits that can be transferred to biological surfaces. *Nat Electron* **1**, 237-245 (2018).
9. Wu P, Reis D, Hu XS, Appenzeller J. Two-dimensional transistors with reconfigurable polarities for secure circuits. *Nat Electron* **4**, 45-53 (2020).
10. Resta GV*, et al.* Doping-Free Complementary Logic Gates Enabled by Two-Dimensional Polarity-Controllable Transistors. *ACS Nano* **12**, 7039-7047 (2018).
11. Pan C*, et al.* Reconfigurable logic and neuromorphic circuits based on electrically tunable two-dimensional homojunctions. *Nat Electron* **3**, 383-390 (2020).
12. Migliato Marega G*, et al.* Logic-in-memory based on an atomically thin semiconductor. *Nature* **587**, 72-77 (2020).
13. Chen H*, et al.* Logic gates based on neuristors made from two-dimensional materials. *Nat Electron* **4**, 399-404 (2021).
14. Liu C*, et al.* Small footprint transistor architecture for photoswitching logic and in situ memory. *Nat Nanotechnol* **14**, 662-667 (2019).
